# 

**Published:** 1873-01

**Authors:** 


### CONTENTS.

**COMMUNICATIONS.**

| | PAGE. |
|---|---|
| A Woman's Right. Ben. H. Pratt, A. M. | 85 |
| Insanities of Sane People. P. H. Hayes, M. D. | 87 |
| Life. Dr. Ned | 89 |

**CORRESPONDENCE.**

| | |
|---|---|
| Nine Letters with Answers | 96 |

**CLIPPINGS.**

| | |
|---|---|
| Lines to a Skeleton—Poetry | 91 |
| How to Soften Hard Putty | 100 |
| Save the Babies | 105 |
| Aids to Digestion | 110 |
| Hygienic Rules | 110 |
| Brain Wasting | 110 |
| The Doctor's Difficulty | 110 |
| Odor of Plants | 110 |

**EDITORIAL.**

| | PAGE. |
|---|---|
| Medical Items and News | 91 |
| Household Hints and Recipes | 98 |
| Time | 101 |
| Tattooing | 101 |
| Reply | 102 |
| Queer Remedies | 102 |
| Fussy | 103 |
| Tear Passages | 104 |
| Editorial Chit-Chat | 106 |
| Books and Periodicals | 111 |

